# Long Non-coding RNAs Involved in Resistance to Chemotherapy in Ovarian Cancer

**DOI:** 10.3389/fonc.2019.01549

**Published:** 2020-01-21

**Authors:** Cecilie Abildgaard, Luisa M. Do Canto, Karina D. Steffensen, Silvia R. Rogatto

**Affiliations:** ^1^Department of Clinical Genetics, Lillebaelt Hospital-University Hospital of Southern Denmark, Vejle, Denmark; ^2^Department of Clinical Oncology, Lillebaelt Hospital-University Hospital of Southern Denmark, Vejle, Denmark; ^3^Institute of Regional Health Research, University of Southern Denmark, Odense, Denmark

**Keywords:** ovarian cancer, lncRNA, drug resistance, chemotherapy, precision medicine

## Abstract

Ovarian cancer (OC) accounts for more than 150,000 deaths worldwide every year. Patients are often diagnosed at an advanced stage with metastatic dissemination. Although platinum- and taxane-based chemotherapies are effective treatment options, they are rarely curative and eventually, the disease will progress due to acquired resistance. Emerging evidence suggests a crucial role of long non-coding RNAs (lncRNAs) in the response to therapy in OC. Transcriptome profiling studies using high throughput approaches have identified differential expression patterns of lncRNAs associated with disease recurrence. Furthermore, several aberrantly expressed lncRNAs in resistant OC cells have been related to increased cell division, improved DNA repair, up-regulation of drug transporters or reduced susceptibility to apoptotic stimuli, supporting their involvement in acquired resistance. In this review, we will discuss the key aspects of lncRNAs associated with the development of resistance to platinum- and taxane-based chemotherapy in OC. The molecular landscape of OC will be introduced, to provide a background for understanding the role of lncRNAs in the acquisition of malignant properties. We will focus on the interplay between lncRNAs and molecular pathways affecting drug response to evaluate their impact on treatment resistance. Additionally, we will discuss the prospects of using lncRNAs as biomarkers or targets for precision medicine in OC. Although there is still plenty to learn about lncRNAs and technical challenges to be solved, the evidence of their involvement in OC and the development of acquired resistance are compelling and warrant further investigation for clinical applications.

## Introduction

Ovarian cancer (OC) is the fifth most lethal cancer in women and accounts for more than 150,000 deaths annually worldwide (1). According to molecular and pathological features, epithelial OCs are stratified into type I or type II (2). Type I OC's (including endometrioid, clear cell, mucinous, and low-grade serous carcinomas) are genetically stable with frequent mutations in *KRAS, BRAF, CTNNB1*, and *PTEN*. In contrast, type II (mainly HGSC) comprises more aggressive tumors with high-grade and propensity for invasion and metastasis leading to high mortality rates (3). These tumors are genetically unstable, presenting a high frequency of *TP53* mutations and *BRCA1/2* alterations. Originally HGSC was thought to arise from the squamous epithelial cell layer of the ovary. However, recent findings demonstrate that the molecular profile of HGSCs has a closer resemblance to the epithelium of the distal fallopian tube, suggesting that this tissue is an alternative site of origin (4, 5). HGSC is the most common and deadliest type of OC and will be the main focus of this review.

Due to the aggressive and invasive nature of HGSC around 70% of the patients have metastatic disease (FIGO stage III-IV) at the time of diagnosis. Surgery combined with chemotherapy is the primary treatment. Platinum-based chemotherapy is the cornerstone of chemotherapeutic treatment, namely cisplatin or carboplatin, combined with a taxane, such as paclitaxel or docetaxel (6). Initially, most patients respond well to the treatment; however, the majority of them will eventually acquire resistance and experience relapse (7, 8). To improve the prognosis, targeted therapies can be applied either as adjuvant or second-line treatments. Bevacizumab, an inhibitor or of vascular endothelial growth factor (VEGF) can be administered as first-line treatment in combination with carboplatin and paclitaxel. Inhibitors of Poly (ADP-ribose) polymerase (PARP) proteins are often used as second-line treatment for recurrent disease, mainly in patients with *BRCA* mutations. A recent randomized phase 3 trial performed in patients with a germline *BRCA* mutation has shown that the addition of oral PARP inhibitor (Olaparib) as maintenance therapy after chemotherapy prolongs the median progression free survival (PFS) by at least 3 years (9).

Despite the comprehensive combination of chemotherapy and maintenance treatment with targeted therapies, most patients develop resistance to treatment. Consequently, patients with disseminated HGSC have an extremely poor prognosis with a 5-year survival rate of only ~20% (10). The knowledge of the underlying molecular mechanisms involved in the development of resistance to chemotherapy is crucial for treatment decisions and the discovery of novel anticancer drug targets.

Advances in sequencing technologies and large-scale genomic projects such as Encyclopedia of DNA elements (ENCODE) (11) and The Cancer Genome Atlas Program (TCGA) (12) have opened avenues to improve our understanding of the mechanisms of response to treatment, development of therapeutic resistance and cancer progression (13–15). Initial studies focused on describing the small percentage of DNA transcribed into RNA encoding for proteins, whereas the non-coding RNA (ncRNA) was regarded as irrelevant and with unknown function for cellular health and disease. However, compelling evidence now reveals the involvement of these transcripts in the regulation of several cellular processes (16, 17). Furthermore, several cancer types have been associated with dysregulated expression of lncRNAs (18).

## LncRNAs in Cancer

NcRNA comprises several different classes of molecules involved in gene regulation and chromatin modification. MicroRNA (miRNA), endogenous small interfering RNA (endo-siRNA) and piwi-interacting RNA (piRNA) are different classes of small ncRNAs involved in heterochromatin formation, histone modification, DNA methylation targeting, and gene silencing. Long non-coding RNAs (lncRNAs) are a subclass of non-translated RNA-sequences defined by an arbitrary length of more than 200 base pairs. These structurally complex RNA molecules interact directly with both DNA, RNA, and proteins affecting various cellular processes including genomic imprinting, gene transcription, mRNA splicing and protein activity (19–21). We are only beginning to understand how these molecules regulate cellular function, and how dysregulation can lead to malignant transformation.

The majority of lncRNAs are physically located in the proximity of protein-coding genes. Furthermore, lncRNAs are often classified according to their position relative to those genes as sense, antisense, intronic, intergenic, and bidirectional (22). Their expression levels are usually low and often compartmentalized to the cytoplasm or nucleus (23). Many lncRNAs exhibit low inter-species homology, and their expression signatures are often tissue-specific, indicating the importance of lncRNAs in cellular differentiation and embryonic development (24–26).

**Figure 1:**
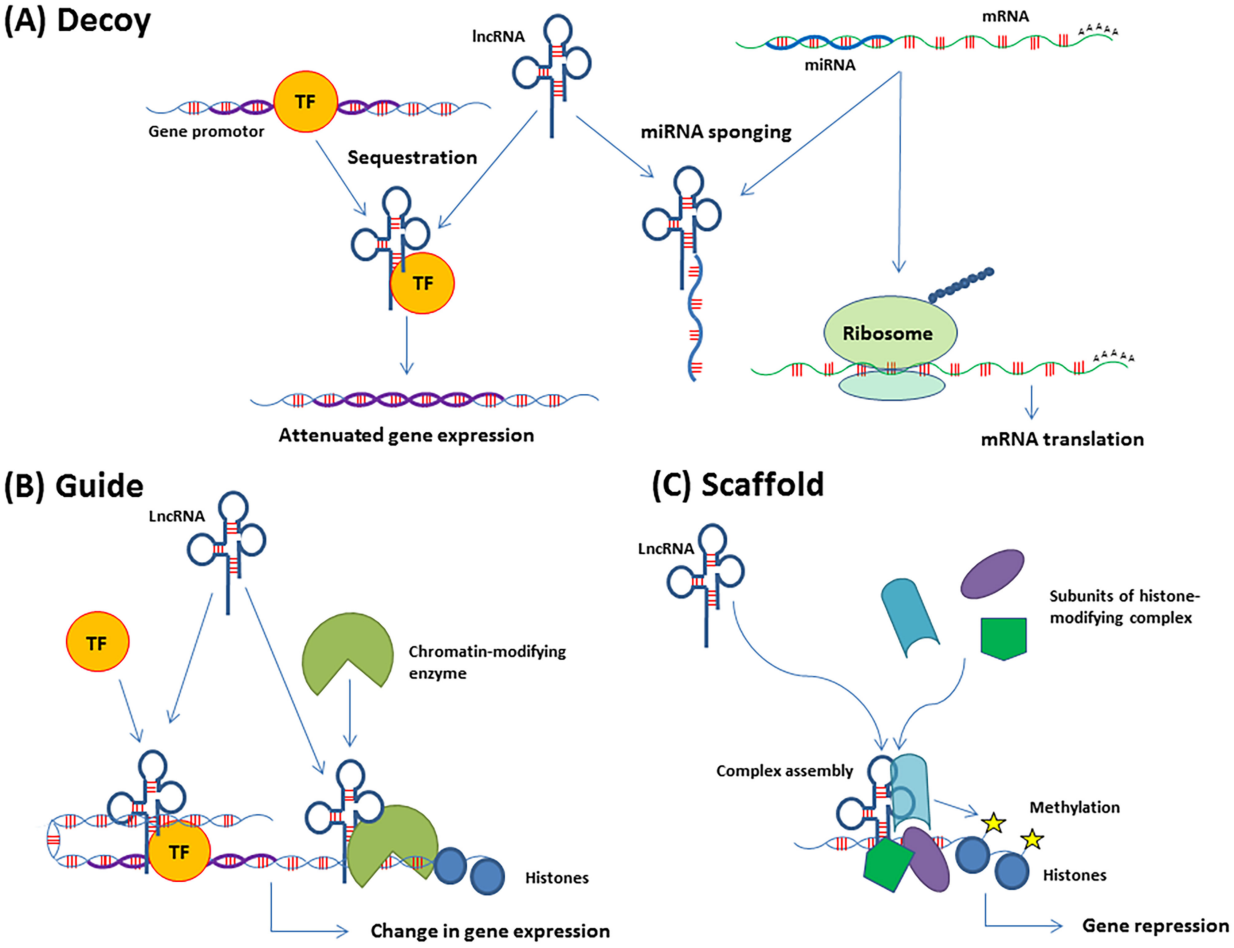
Functions of lncRNAs in gene regulation. **(A)** Decoys can sequester transcription factors (TF) or complementary RNA transcripts, such as miRNAs (also called miRNA sponging). The consequence of TF sequestration is attenuated expression of the genes regulated by that TF. The effect of miRNA sponging is the release of the molecule e.g., mRNA which is targeted by that miRNA. The mRNA is then translated. **(B)** Guides recruit molecules, such as TFs or chromatin-modifying enzymes to their target areas of the genome, which leads to the regulation of gene expression. **(C)** Scaffolds support transient assembly of protein complexes at genomic regions, which can promote histone modifications and DNA methylation.

The lncRNA mechanisms of action usually fall into three categories, decoys, guides, or scaffolds (Figure 1). The decoys function as competing endogenous RNAs (ceRNAs) and modulate gene expression by sequestering transcription factors or miRNAs (also called sponging). Consequently, the availability of the targeted molecule is limited and the downstream effect reduced. The guide lncRNAs help to localize transcription factors or chromatin modifiers to specific areas of the genome, whereby transcription can be modulated. Dynamic scaffolds support transient assembly of protein complexes that bind genomic regions to affect chromatin structure (27, 28). The functions are not mutually exclusive, and many lncRNAs have more than one function. The single-stranded circular RNA (circRNA) is a sub-group of lncRNA recently discovered (29). Although the function of circRNA is still poorly understood, evidence indicates a role in miRNA regulation by sponging and intracellular transportation. LncRNAs are also stratified into *cis*- and *trans*-acting regulators, where the *cis*-regulators, exert their effect on neighboring genes on the allele from which they are transcribed, and the *trans*-regulators control gene expression at distant genomic sites.

Unsurprisingly, aberrant expression of lncRNAs has been associated with several diseases, including cancer. Dysregulated lncRNAs can exert oncogenic or tumor suppressor functions through transcriptional regulation impacting cellular proliferation, differentiation, invasiveness, apoptosis, and metabolism (30).

Many cancer-associated lncRNAs display similar expression patterns in different cancer types. Overexpression of Hox transcript antisense RNA (HOTAIR) was first described in breast cancer, where it was associated with increased invasiveness and metastasis (31). Subsequent studies revealed an association of increased expression of *HOTAIR* with disease progression and poor prognosis in colorectal (32), non-small cell lung (33), hepatocellular (34–36), gastric (37, 38), pancreatic (39), and ovarian (40) carcinomas. Single nucleotide polymorphisms (SNPs) in *HOTAIR* were recently correlated with increased susceptibility to develop OC in a Chinese population (41, 42). In ovarian cancer, *HOTAIR* overexpression was associated with poor differentiation, advanced FIGO stage and lymph node metastasis (40, 43).

Metastasis associated lung adenocarcinoma transcript 1 (MALAT1) is another lncRNA widely overexpressed in various solid tumor types (44, 45), including OC (46–49). Several studies on OC cell lines showed that depletion of *MALAT1* suppresses viability, proliferation, migration, and invasion (46, 47, 50). *MALAT1* is highly conserved among mammals and is primarily known to localize to nuclear splicing speckles, where it interacts with splicing factors to regulate alternative splicing (51). In OC, *MALAT1* was demonstrated to suppress alternative splicing of pro-apoptotic factors, causing apoptotic and anoikis resistance (50).

*HOTAIR* and *MALAT1* are examples of widely expressed lncRNAs with oncogenic potential. Several other well-studied lncRNAs are found to be involved in the regulation of cellular processes such as proliferation, genomic stability, metabolism, and apoptosis to ensure homeostasis. These functions are executed through the lncRNAs directly or indirectly influence on the transcription of various proteins, which can lead to context-dependent oncogenic or tumor-suppressive properties. For a comprehensive overview of lncRNA's involved in cancer see (52–54).

A better understanding of the interplay between coding- and non-coding RNA and the integration of more molecular markers could potentially improve the predictive value of the molecular subtypes and provide a stronger tool for personalized therapeutic approaches.

## The Molecular Landscape of HGSC

The most prominent molecular feature of HGSC is high genomic instability (55), possibly initiated by *TP53* dysregulation and its associated effects in DNA damage repair (56). Mutations of *TP53* were reported in up to 96% of HGSC cases, mostly missense mutations (70.4%), which can result in a dominant-negative effect, gain or loss of protein function. Frameshift (12%), non-sense (8.67%), and splice site (5%) mutations, leading to loss of protein function have also been described (57). Only a few other genes were reported as commonly mutated in HGSC, including *BRCA1* (12.5%) and *BRCA2* (11.5%) (58).

Genetic predisposition is recognized in a minority of the patients with HGSC, with around 70% of familial cases presenting inherited pathogenic mutations in *BRCA1* and *BRCA2* (59). These mutations contribute to an increased risk of developing ovarian cancer (44% for *BRCA1* and 27% for *BRCA2* carriers), compared to the normal population. Mutations in other genes with low penetrance also have an important role in ovarian cancer development. The increased lifetime risk for women harboring mutations in genes involved in the DNA damage repair by homologous recombination (HR), such as *BRIP1* (5.8%) (60), *RAD51C* (5.2%), and *RAD51D* (12%) have been reported in OC (61). Alterations in genes involved in DNA mismatch repair associated with Lynch syndrome (*MSH2, MLH1, PMS2*, and *MSH6*), in rare cases prompt HGSC development (59, 62, 63).

The deficiency in DNA damage repair pathways is compatible with the high genomic instability observed in epithelial OC, with copy number alterations (CNA) affecting a significant fraction of the genome. Recurrent focal amplification of *CCNE1, MYC*, and *MECOM* genes are frequently identified in the TCGA cohort (58). Cases showing *CCNE1* amplification are mutually exclusive with *BRCA* mutated cases suggesting the involvement of different pathways in the tumorigenesis of HGSC (58). Deficiency of the HR pathway was described in around 50% of HGSC cases, which has been associated with *BRCA1* (20% of cases) and *BRCA2* (5%) germline or somatic mutations and, *BRCA1* promoter hypermethylation (10%). Genomic alterations in other genes involved in the HR repair pathway, such as amplification or mutation of *EMSY* (8%), focal deletion or mutation of *PTEN* (7%), hypermethylation of *RAD51C* (3%), mutation of *ATM/ATR* (2%), and mutation of Fanconi Anemia genes (58) have also been reported.

Some sporadic ovarian tumors share the phenotypic traits with tumors harboring germline mutations in *BRCA1/2* (BRCAness phenotype), which may reflect molecular similarities. The BRCAness phenotype predicts responsiveness to platinum-based chemotherapy (64) and PARP inhibitors (65). In a population-based study that evaluated the mutational profile of HR genes, a better overall survival in BRCAness patients was described (66). Another approach to identifying HR deficiency was performed based on scores of the CNA profile of tumors, named “genomic scars,” which was very high in HGSC, and also correlated to PARP inhibitors or platinum-based chemotherapy sensitivity (67).

The integrative analysis of CNA, mutations, and gene expression alterations of HGSC identified RB1 and PI3K/Ras pathways deregulated in 67% and 45% of the cases, respectively (58). Amplification of *PIK3CA, PIK3CB*, and *PIK3K4* was correlated to the decreased overall survival of OC patients. The analysis of *PIK3CA* protein product p110α and p-Akt confirmed the involvement of the PIK3/AKT pathway in survival (68). The PIK3A/AKT/mTOR pathway was also shown to be implicated in therapy resistance. Advanced OC patients who did not respond to subsequent chemotherapy presented activation of the pathway compared to responsive patients (69). Besides, *GAB2*, a signaling intermediate of PI3K and MAPK pathways, was reported as amplified in 44% of ovarian cancer samples (70). Although HGSC rarely exhibits mutations in *KRAS* or *BRAF*, the main activators of the MAPK pathways, almost half of tumors display an expression of active downstream MAPKs (71).

In addition to the specific pathways and genes altered in HGSC, distinct molecular subtypes were identified based on the differential expression profiles (72). The expression analysis of 489 tumors performed by TCGA and compared to an external cohort revealed four HGSC subtypes: proliferative, mesenchymal, immunoreactive, and differentiated (58). The proliferative subtype was characterized by low expression of ovarian tumor markers and high expression of transcription factors and proliferation markers. The mesenchymal subtype presented a high expression of *HOX* genes and markers suggestive of increased stromal components. The immunoreactive subtype was characterized by T-cell chemokine ligands, *CXCL11* and *CXCL10*, and the receptor, *CXCR3*. The differentiated subtype was related to high expression of *MUC16, MUC1*, and *SLPI* (the secretory fallopian tube maker), suggesting a more mature stage of development (58). Patients with the HGSC immunoreactive subtype presented better prognosis, while patients with the mesenchymal or proliferative subtypes showed worse overall survival (73, 74).

## LncRNAs Signature of Ovarian Cancer

Lately, the predictive value of differentially expressed lncRNAs has received increased attention due to their presence in liquid biopsies and potential as biomarkers for therapeutic response and prognosis (49, 75, 76). A meta-analysis including 1,333 OC patients established that altered lncRNAs are, in general, associated with decreased overall survival (76). In this analysis, 11 lncRNAs (*HOTAIR, TC010441, AB073614, ANRIL, MALAT1, NEAT1, CCAT2, UCA1, HOXA11-AS, SPRY4-IT1*, and *ZFAS1*) were identified with significantly increased expression in OC patients (76). Additional data have been reported to support the role of lncRNAs in OC. Eight lncRNAs were significantly correlated with overall survival, in a comprehensive analysis of lncRNA expression profiles of 544 OC patients from TCGA (75). Six of them (*RP4-799P18.3, RP11-57P19.1, RP11-307C12.11, RP11-254I22.1, RP1-223E5.4, and GACAT3*) were positively correlated with overall survival, while the last two (*PTPRD-AS1 and RP11-80H5.7*) were inversely correlated. The eight-lncRNA signature showed prognostic value and was able to stratify patients according to clinical outcome into high- and low-risk groups. Furthermore, this signature demonstrated predictive value for the response to platinum-based chemotherapy (75). A prognostic signature was identified for recurrent disease based on datasets extracted from the Gene Expression Omnibus (GEO). The signature comprised four well-known cancer-related lncRNAs, *RUNX1-IT1, MALAT1, H19*, and *HOTAIRM1*, and two less well-described transcripts *LOC100190986* and *AL132709.8*. These lncRNAs were confirmed as differentially expressed in validation cohorts independently of tumor stage, tumor grade and histology type (49).

*In silico* analysis of RNA sequencing data derived from 391 patients retrieved from TCGA revealed three additional lncRNAs (*NBR2, ZNF883, and WT1-AS*) associated with recurrent OC (77). Based on the results, two interesting interactions were predicted; *WT1-AS-miR-375-RBPMS* and *WT1-AS-miR-27b-TP53*, suggesting that *WT1-AS* regulates two important tumor suppressors RBPMS and *TP53*, through miRNA sponging (77).

**Figure 2:**
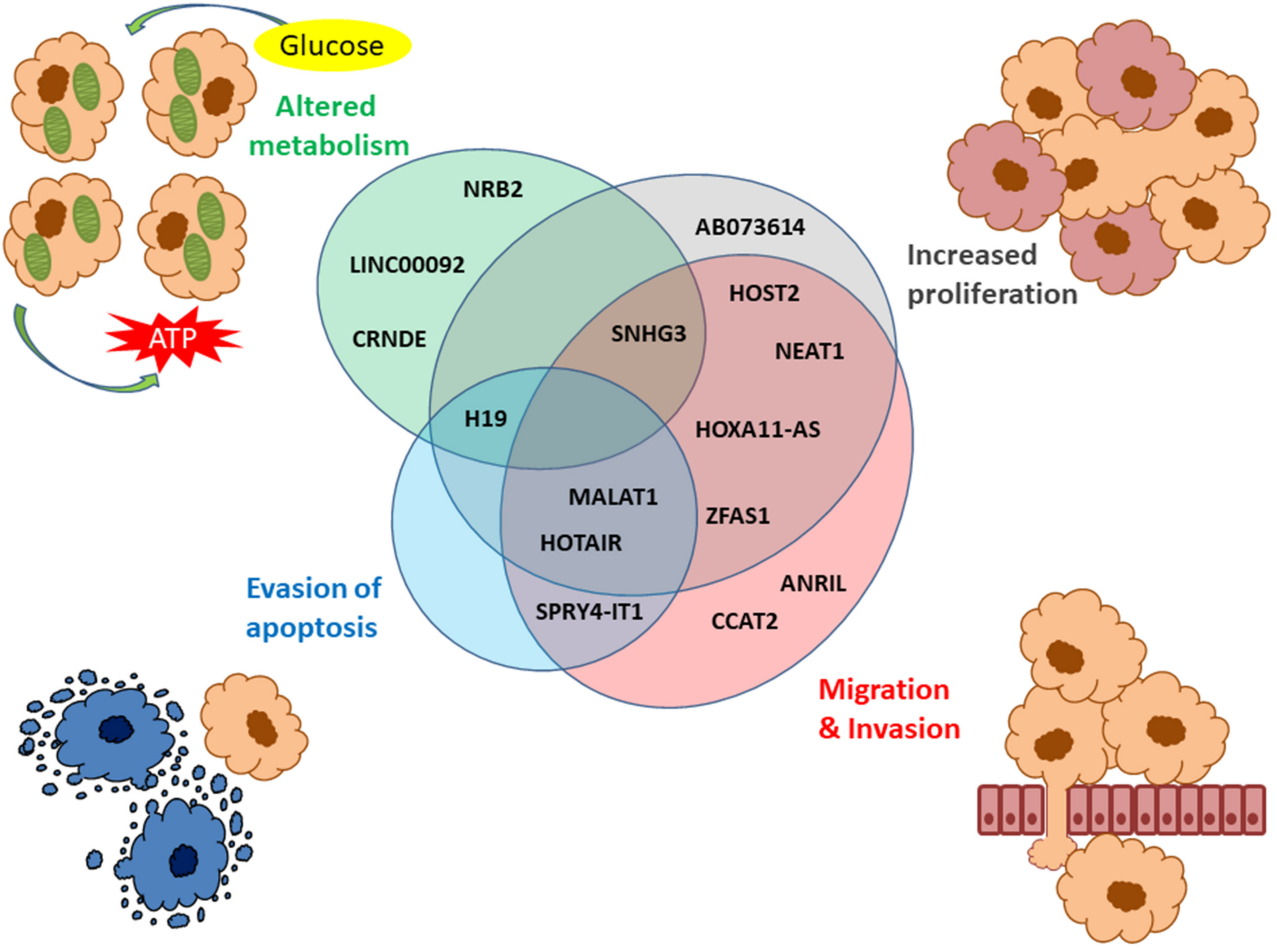
LncRNAs described in OC associated with the hallmarks of cancer. The hallmarks of cancer include increased proliferation (gray), migration/invasion (red), evasion of apoptosis (blue), and altered metabolism (green). The Venn diagram shows the lncRNAs associated with these four hallmarks with several of them involved in more than one hallmark of cancer.

Several lncRNAs identified in large-scale studies have also been validated individually (46, 78–82), and across different cancer types (83). Also, functional studies have revealed that many of the dysregulated lncRNAs associated with OC are involved in one or several hallmarks of cancer such as increased proliferation, altered metabolism, evasion of apoptosis, migration or invasion (Figure 2) (79, 84–86).

Two lncRNAs are described specifically in ovarian cancer, ovarian adenocarcinoma amplified (OVAL) (87) and human ovarian cancer-specific transcript 2 (HOST2) (88). An intergenic region encompassing the full *OVAL* gene was found amplified in higher frequency in OC patients in comparison to other cancers. *OVAL* amplification and its increased expression suggest an oncogenic function in OC (87). So far no mechanisms or functional interactions have been described for *OVAL*. The expression of *HOST2* is dramatically increased in OC tissues and cell lines, compared to normal ovarian tissues and non-ovarian cell lines (88). Furthermore, *HOST2* was associated with increased proliferation, migration and invasion in OC. The mechanism of action of this potential driver is suggested to be through sequestration of miRNA let-7b, which is known to promote the expression of several oncogenes (89).

The involvement of dysregulated lncRNAs in the development of OC is well-documented. Considering the described oncogenic and tumor suppressor functions of lncRNA, their role in the development of resistance to therapy is expected (90–92). Differential lncRNA expression profiles were demonstrated in cisplatin-resistant and cisplatin sensitive OC, supporting their role in acquired resistance to chemotherapy (93). However, the mechanism by which lncRNAs contribute to acquired resistance remains incompletely understood. More detailed insights might lead to discoveries of new biomarkers or therapeutic targets. Signatures with the potential to predict therapeutic resistance would be valuable tools for clinicians, aiding the selection of the optimal treatment strategies for individual patients. In the following section, the involvement of lncRNAs in the development of therapeutic resistance will be highlighted, with a focus on platinum and taxane-based treatment regimens.

## LncRNAs Involved in Platinum-Resistance

The development of anti-cancer drug resistance is often complex and multifactorial, depending on the specific drug and the histological subtype of cancer. Carboplatin and cisplatin are the most commonly used drugs for the first-line treatment of advanced stages of HGSC. These platinum-based agents interact with DNA forming mono adducts or interstrand, intrastrand, and protein crosslinks mainly at guanine. The crosslinking affects DNA repair and synthesis and leads to the accumulation of single and double-strand breaks, which results in cell cycle arrest and apoptosis (94, 95). Moreover, the release of reactive oxygen species that activates inflammatory pathways may also contribute to the cytotoxic effects of these compounds (96).

Resistant clones can arise through a clonal selection of cells able to prevent, repair, or withstand DNA damage. The tumor suppressor p53 and its related nuclear transcription factors are important mediators of the cytotoxic effects of platinum therapy. DNA damage normally leads to a p53-dependent release of pro-apoptotic factors. Consequently, reduced activity of p53 or the related pathways is associated with platinum-resistance (97). Platinum-induced DNA damage can also be repaired by HR, and hence, the activity of the *BRCA1/2* genes reduces the responsiveness to platinum therapy (98). In accordance, reversions of *BRCA1/2* germline mutations have previously been reported as a mechanism of resistance to therapy (8). The mismatch repair system is another mechanism by which the cell can detect platinum induced lesions. Loss of mismatch repair-related genes such as *MLH1* and *MSH2* prevents the cells from recognizing the damage caused by platinum therapy. Consequently, apoptosis is not initiated and the cells are therefore less sensitive to the treatment (99, 100). Furthermore, platinum resistance was associated with epithelial to mesenchymal transition (EMT) (101), implying a range of molecular alterations promoting invasive properties and resistance to oncogene-induced senescence. The major pathways involved in EMT are TGF-β, HIF1-α, Wnt/β-catenin, and Notch, which have also been associated with platinum resistance (102, 103). General resistance mechanisms include reprogramming of metabolism and mitochondrial dysregulation, suppression of apoptotic mediators, up-regulated efflux pumps, reduced drug uptake, or intracellular detoxification (104). The molecular alterations leading to the platinum-resistant phenotype rarely include single nucleotide mutations in the known driver or resistance genes. Instead, it appears that the resistance arises from a highly patient-specific and adaptable pattern of altered methylation, gene amplifications, reversion of *BRCA1/2* mutations, promotor fusion, and translocation, and differential expression of ncRNAs (8, 105, 106).

**Figure 3:**
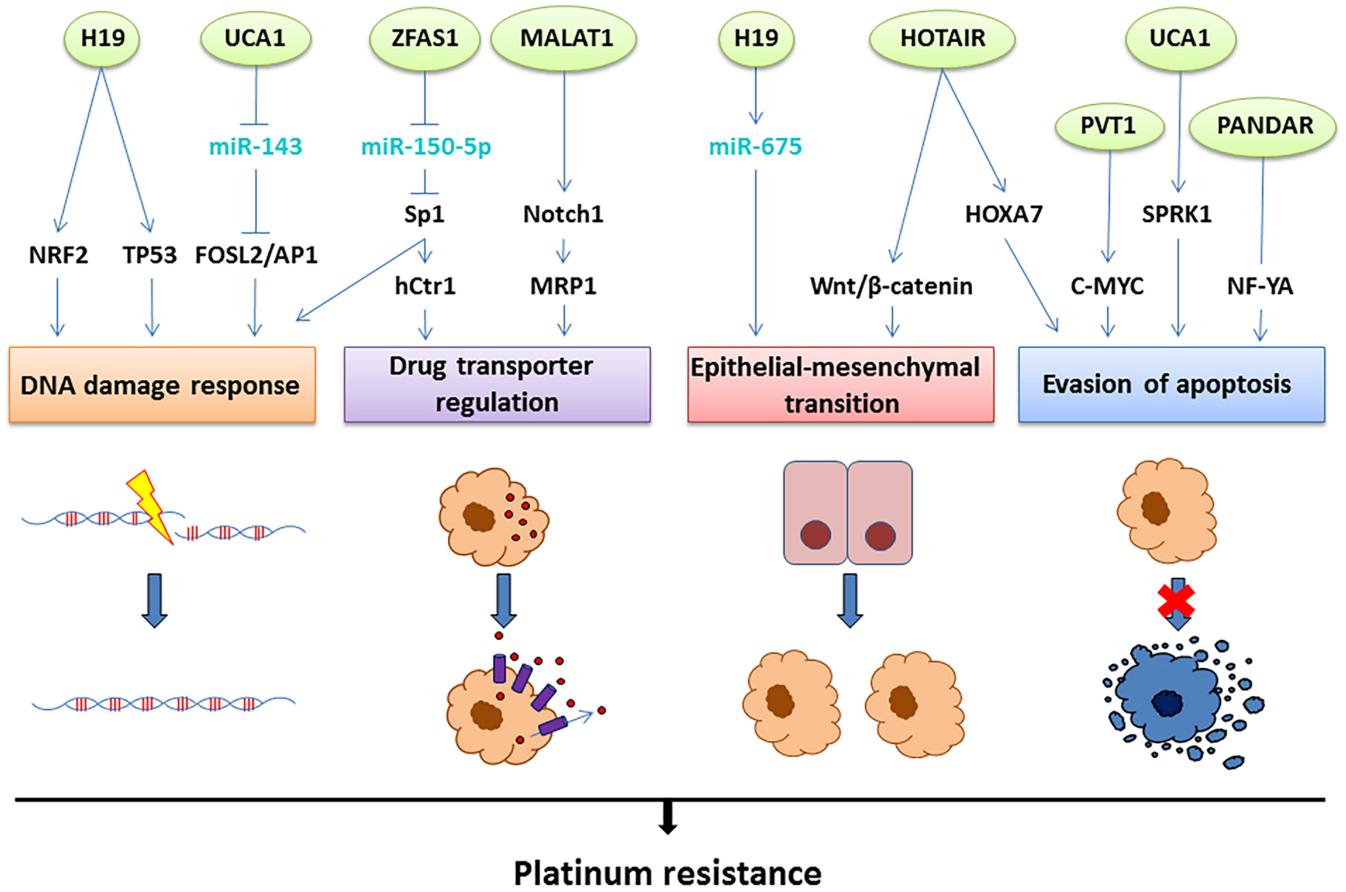
The lncRNAs involved in platinum resistance in OC. Aberrant expression of the lncRNAs depicted on top (green circles) leads to platinum resistance through four main mechanisms: improved DNA damage response, upregulation of drug transporters leading to efflux of the drug, epithelial-mesenchymal transition or evasion of apoptosis. The molecular mechanisms suggested linking the lncRNAs to these resistance mechanisms involve interactions with miRNAs (light blue) and direct or indirect regulation of transcription factors and signaling pathways (black), as illustrated above.

Although several lncRNAs have been associated with platinum resistance in OC (Table 1), the resistance-associated molecular mechanisms have been elucidated in only a few of them (Figure 3). Next, we will present the current knowledge of the functions and roles of a set of lncRNAs associated with platinum resistance in OC.

**Table 1:** List of lncRNAs associated with platinum-resistance in ovarian cancer.

**lncRNA**	**Category**	Expression in OC tissue^*^	Expression in platinum-resistant cell lines^**^	Mechanisms of resistance^***^	**References**
*HOTAIR*	Antisense	↑ (cisplatin resistance or treated with carboplatin)	↑ (cisplatin/carboplatin)	↑ *HOXA7*	(107–110)
*MALAT1*	Intergenic	↑	↑ (cisplatin)	↑ Notch1 → ↑ abcc1	(111, 112)
*H19*	Intergenic	↑ (recurrent disease)	↑ (cisplatin)	↑ GSH pathway	(113)
*ZFAS1*	Antisense	↑ (platinum resistance)	↑ (cisplatin)	↓ miR-150-5p → ↑ SP1	(80, 114)
*UCA1*	Intergenic	↑ (cisplatin resistance)	↑ (cisplatin)	↑ SRPK1 ↓ miR-143 → ↑ FOSL2	(115–117)
*PANDAR*	Antisense	↑ (disease recurrence + wt-p53)	↑ (cisplatin)	↓ NF-YA ↑ SFRS2 - ↓ p53	(118)
*PVT1*	Intergenic circRNA	↑ (cisplatin resistance)	↑ (cisplatin)	↑ c-MYC	(119)
*ZBED3-AS1*	Antisense	↑ (platinum resistance)	↑ (cisplatin/carboplatin)	N/A	(120)
*F11-AS1*	Antisense	↓ (platinum resistance)	↓ (cisplatin/carboplatin)	N/A	(120)
*GAS5*	Intergenic	↓ (platinum resistance)	↓ (cisplatin/carboplatin)	N/A	(120)
*BC200*	Intergenic	↓	↓ (carboplatin)	N/A	(121)
*LINC00312*	Intergenic	↓ (cisplatin+paclitaxel resistance)	↓ (cisplatin)	↑ Bcl-2/Caspase-3 pathway	(122)
*BX641110*		N/A	↑ (cisplatin)	N/A	(123)
*CRNDE*	Intergenic	N/A	↑ (cisplatin)	N/A	(123)
*HOXC-AS3*	Antisense	N/A	↑ (cisplatin)	N/A	(123)
*RP11-384P7.7*	Intergenic	N/A	↑ (cisplatin)	N/A	(123)
*PLAC2*	Intronic	N/A	↑ (cisplatin)	N/A	(123)
*RP11-6N17*	Intergenic	N/A	↑ (cisplatin)	N/A	(123)
*RP11-65J3.1-002*	Intergenic	N/A	↓ (cisplatin)	N/A	(123)
*AC141928.1*	Intergenic	N/A	↓ (cisplatin)	N/A	(123)
*GS1-600G8.5*	Intergenic	N/A	↓ (cisplatin)	N/A	(123)
*SNHG15*	Intergenic	↑	↑ (cisplatin)	N/A	(124)
*EBIC*	Processed pseudogene	↑	↑ (cisplatin)	↑ Wnt/β-catenin pathway	(125)

*The order of the lncRNAs corresponds to the appearance of the individual descriptions in the manuscript. Only the first 7 lncRNAs are described in detail in the manuscript, and are selected based on substantiating evidence in the literature*.

* *The expression of the lncRNAs in OC tissue is indicated by arrows, ↑ for higher and ↓ for lower expression in platinum-resistant patients (patient characteristics indicated in parenthesis), compared to expression in platinum-sensitive patients. If no patient characteristics are indicated, the expression was determined in ovarian cancer tissue from patients with unspecified sensitivity to platinum drugs and normalized to adjacent or normal ovarian tissue*.

** *The expression in resistant OC cell lines is indicated by arrows; ↑ for higher and ↓ for lower expression in platinum-resistant, compared to expression in platinum-sensitive cell lines. The drug the cell lines are resistant to is indicated in parenthesis*.

*** *The effect of lncRNAs on associated pathways, miRNAs, genes or transcription factors involved in resistance mechanisms are indicated by arrows: ↑ induction and ↓ repression*. *N/A, information not available*.

*HOTAIR* located within the *HOXC* gene cluster (mapped on12q13.13) was previously introduced due to its involvement in OC. *HOTAIR* recruits lysine-specific demethylase 1 (LSD1) and Polycomb Repressive Complex 2 (PRC2) and guide them to promote epigenetic silencing of *HOXD* genes (126). Additionally*, HOTAIR* regulates other HOX genes including *HOXA7*, which is consistently overexpressed in several tumor types (40, 127, 128). The knockdown of *HOTAIR* led to reduced expression of *HOXA7*, which increased susceptibility to apoptosis and restored cisplatin sensitivity in resistant OC cells (107). In general, *HOTAIR* is more abundant in advanced OC tissues and was also overexpressed in cisplatin-resistant OC cell lines, compared to sensitive controls (108). Furthermore*, HOTAIR* expression was correlated with poor survival in patients who received carboplatin compared with untreated patients (109). Knockdown of *HOTAIR* in a mouse xenograft model, enhanced the effect of treatment with cisplatin, suggesting its potential as a target to re-sensitize ovarian cancer cells to platinum treatment. This effect has been attributed to reduced activation of the Wnt/β-catenin pathway, which is known to promote excess stem cell renewal and EMT (110). Thus, overexpression of *HOTAIR* might contribute to platinum resistance by increased transcription of *HOXA7* and Wnt/β-catenin dependent induction of EMT. Overexpression of three additional lncRNAs *DNM3OS, MEG3*, and *MIAT* have been associated with EMT in ovarian cancer (129). However, the direct link between dysregulation of these transcripts and the development of platinum resistance is unexplored.

As previously mentioned, *MALAT1* plays an important oncogenic role in multiple cancers (44, 45). Recently, *MALAT1* has also been associated with resistance to therapy (130). In OC, *MALAT1* knockdown increased cell death during treatment with cisplatin, indicating its potential involvement in resistance (111). *MALAT1* was demonstrated to correlate with NOTCH1 expression, which is also up-regulated during platinum resistance in OC (112). NOTCH1 knockdown attenuates cisplatin resistance by directly down-regulating the expression of the multidrug resistance-associated protein 1 (*ABCC1*) in OC (131). *ABCC1* encodes a transporter of molecules across cellular membranes, including the efflux of a range of drugs (132). In lung adenocarcinoma and colorectal cancer*, MALAT1* promotes resistance to taxane- and platinum-based drugs, respectively. In these cases, EMT was identified as a mechanism of resistance (130, 133); however, this effect has not yet been investigated in OC.

The imprinted maternally expressed transcript *H19* gene is located in a well-conserved gene cluster also containing the insulin-like growth factor 2 (IGF2). Both genes are regulated by genomic imprinting, and *H19* is only transcribed from the maternal allele, whereupon it plays an important role in embryonic development and growth control (134). Aberrant expression of *H19* has been demonstrated in several different cancers (135, 136); although its exact carcinogenic role is still under debate (137). The understanding of its function is challenged by the variety of transcriptional products deriving from the *H19* gene locus and its complex regulation. As an example, the miR-675 is transcribed from the first exon of *H19* (138) and has been associated with EMT and metastatic progression in colorectal and pancreatic cancers (139, 140). Furthermore, *H19* is directly induced by the c-Myc oncogene (141) and its expression has been associated with the hypoxic stress response, involving p53 and hypoxia-inducible factor 1-α (HIF1-α) (142). Transcriptome analysis revealed differential expression of *H19* in cisplatin-resistant OC cells compared to their sensitive progenitors. The involvement of *H19* in platinum resistance was validated in tissues from 41 cases of HGSC treated with either cisplatin or carboplatin. The patients were divided into two groups according to their recurrence-free survival (threshold of 12 months), where *H19* expression was shown to be significantly higher in patients with early recurrence (113). The role of *H19* in cisplatin resistance was related to oxidative stress and induction of the glutathione (GSH) pathway, where *H19* regulates several targets (*GSR, G6PD, GCLC, GCLM, GSTP1*, and *NQO1*) of the nuclear factor erythroid 2 (*NRF2)*, an important factor in the antioxidant defense (113). The glutathione pathway has been suggested as a detoxifying mechanism to platinum-induced oxidative toxicity and is often up-regulated during the development of resistance (143, 144). *H19* overexpression has also been correlated with cisplatin resistance in other cancers, including seminomas (145) and non-small cell lung cancer (146), where it was associated with evasion of apoptosis.

ZNFX1 antisense RNA 1 (ZFAS1) is transcribed from the antisense strand close to the protein-coding gene *ZNFX1*, including three C/D box small nucleolar RNAs (Snord12, Snord12b, and Snord12c) (147). The role of the lncRNA *ZFAS1* (zinc finger antisense 1) varies among human cancers. *ZFAS1* is downregulated in breast tumors compared to normal mammary tissue (147), whereas it is overexpressed in colorectal cancer (148), indicating tissue-specific functions. In OC, *ZFAS1* overexpression was identified as part of an eight-lncRNA expression signature predictive of platinum-sensitivity, based on transcriptome data from 258 patients with HGSCs (114). The authors also found increased *ZFAS1* expression in cisplatin-treated OC cell lines. Functional studies in OC cell lines revealed that the *ZFAS1* knockdown resulted in increased sensitivity to cisplatin. This effect was shown to involve sequestration of miR-150-5p, which prevents binding of the transcription factor specific protein 1 (SP1) (80). *SP1* has been appointed an important oncogene and a potential therapeutic target in several tumor types (149). Additionally, *SP1* is involved in DNA damage response (150), and regulation of a copper transporter (hCtr1), which is associated with platinum drug transport (151).

Urothelial carcinoma associated 1 (UCA1) is expressed during embryonic development and subsequently abolished in most tissues, including ovarian epithelia. In OC tissue and several other cancers, *UCA1* is re-activated and overexpressed (115, 152). In cancer tissues, *UCA1* is regulated by HIF1-α, indicating its involvement in the response to hypoxia (153). Through sponging of miR-143, *UCA1* prevents the repression of FOSL2, a subunit of the Activator protein 1 (AP-1) also involved in the hypoxic regulation. Consequently, *UCA1* overexpression might lead to the up-regulation of the hypoxic response involving AP-1. Hypoxia has been shown to promote cisplatin resistance, through HIF1-α and p53 activation (154). A significant *UCA1* overexpression in OC tissues from cisplatin-resistant patients (116) was reported. *In vitro* assays revealed that stable transfection of OC cells with *UCA1* promotes resistance toward cisplatin (115). In addition, *UCA1* was shown to affect the activity of the serine/arginine-rich protein-specific kinase 1 (SRPK1), an oncogene that suppresses apoptotic factors (115).

*UCA1* is also associated with resistance to Paclitaxel in OC (155, 156), which can be reverted by *UCA1* knockdown in cell lines (156). This effect was related to reduced sponging of miR-129 and, subsequently down-regulation of the *ABCB1* gene that encodes an efflux pump previously correlated with multidrug resistance in cancer (156, 157). These findings suggested that the mechanism of resistance involving *UCA1* is multifactorial, and could include improved response to DNA damage, reduced activation of apoptotic factors and increased efflux of the drugs.

The promoter of *CDKN1A* Antisense DNA damage Activated RNA (PANDAR) is a widely acknowledged oncogene mainly involved in regulating the response to DNA damage (158). The transcription of *PANDAR* is p53-dependent and promotes cell survival by impeding apoptosis through sequestering of the NF-YA transcription factor (159). In OC cell lines, an inverse relationship was demonstrated between *PANDAR* expression and cisplatin sensitivity. This effect involved interaction between *PANDAR* and the splicing factor arginine/serine-rich 2 (SFRS2), which led to negative feedback regulation of *TP53* (118). Patients with wild type *TP53* showed increased expression of *PANDAR* and SFRS2 at disease recurrence, compared to the time of diagnosis (118). The *PANDAR*-dependent suppression of p53 in resistant cells prevents the normal DNA damage response, whereby the cells can evade apoptosis. Since HGSCs have a very high occurrence of inactivating *TP53* mutations, the role of *PANDAR* in platinum resistance remains to be elucidated.

Plasmacytoma variant translocation 1 (PVT1) is a well-established oncogene in OC (160, 161), as well as other cancers such as gastric (162) and breast (163). *PVT1* is located in proximity to the *MYC* locus, where it encodes several alternative splice isoforms. In addition, the *PVT1* locus contains a cluster of at least six miRNAs (miR-1204,−1205,−1206,−1207-3p,−1207-5p, and−1208) (164). Transcription of *PVT1* can be regulated by p53 through a canonical binding site, indicating its involvement in the response to DNA damage. *PVT1* is often co-expressed with *MYC*, with which it interacts and stabilizes to potentiate its activity (165). *MYC* has been suggested as a potential therapeutic target in platinum-resistant OC, as its overexpression confers resistance toward cisplatin (166). The role of *PVT1* in the development of therapeutic resistance in OC is ambiguous since it was both demonstrated to promote cisplatin resistance by suppressing apoptotic factors (119), but also to be an effector in the cytotoxic response to treatment with carboplatin and docetaxel, by activating p53 and potentially promoting apoptosis (167). However, since p53 is often affected by the loss of function mutations in HGSC, the cytotoxic effect of *PVT1* in response to carboplatin and docetaxel might be blunted in these cases. Studies in other cancers support the involvement of *PVT1* in cisplatin resistance (168, 169). The opposing effects described for *PVT1* could be due to the differences in the mechanisms of action of the two treatment regimens and underlines the need for further investigation.

Other mechanisms of resistance than the ones reported here have been suggested to involve lncRNAs. As an example, the lncRNAs *MPRL* (170), *LINC00312* (122), and *SNHG3* (84) are involved in mitochondrial function and altered expression of these have been associated with platinum resistance, either through effects on energy metabolism or mitochondrial-dependent apoptosis. In general, the interactions between lncRNA and the mitochondrial genome is not yet well-understood and should be further investigated.

## LncRNAs Involved in the Taxane Resistance

Taxanes are microtubule-stabilizing agents that bind to the β-subunit of tubulin dimers to promote and stabilize polymerization. This mechanism inhibits microtubule disassembly that is a necessary event in mitosis; consequently, mitotic arrest and eventually apoptosis are promoted (171, 172). Paclitaxel is most often used in combination with the platinum-based chemotherapy as a first-line treatment, or as a single agent in platinum-resistant OC patients. Unfortunately, repeated exposure often leads to acquired resistance. Docetaxel, a second-generation taxane, can be used in some cases; however, shared resistance mechanisms result in low response rates. *In vitro* experiments have demonstrated that an inverse relationship exists between resistance to platins and taxanes, suggesting separate resistance mechanisms and emphasizing the benefits of combined treatments (173). The most common resistance mechanisms to taxanes comprise structural changes in the β-tubulin target region, altered expression of apoptotic and mitotic factors and overexpression of the multidrug resistance genes (ABC-transporters) (174, 175).

Since the taxanes are rarely used as a single agent in the treatment of OC, only a few studies have investigated the role of lncRNAs in the development of paclitaxel resistance in tissues. A combined analysis of two expression datasets comparing (1) patients with complete and incomplete response to chemotherapy and (2) two OC cell lines with paclitaxel resistance with two sensitive OC cell lines was performed. The combined analysis identified a panel of seven lncRNAs (*XR_948297, XR_947831, XR_938728, XR_938392, NR_103801, NR_073113*, and *NR_036503*) differentially expressed in both cell lines and tissues, and had predictive value for resistance to therapeutic regimens containing paclitaxel (176). However, the signature described in this study needs further validation and the functional implications for differential expression of the selected lncRNAs should be explored.

**Figure 4:**
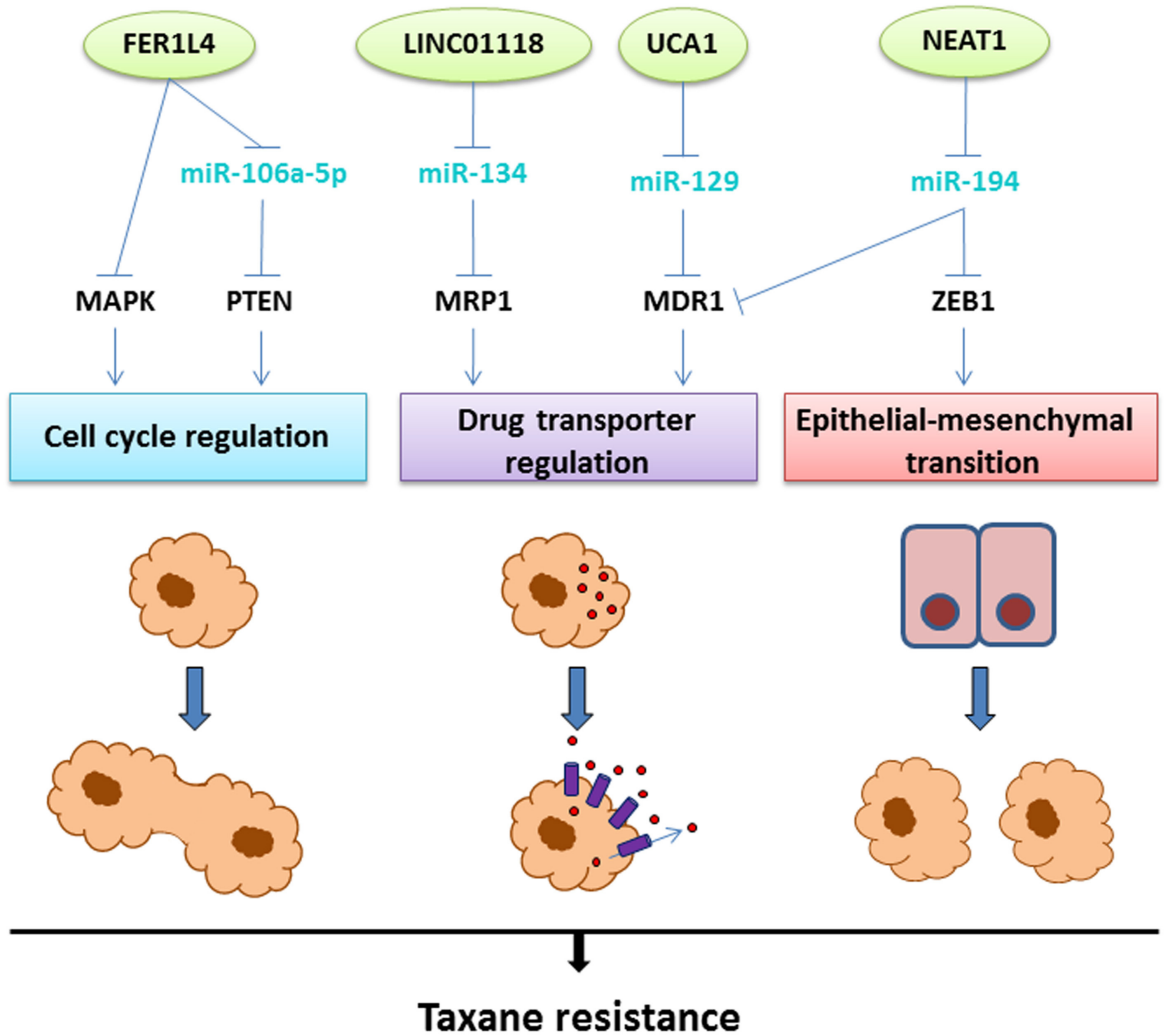
The lncRNAs involved in taxane resistance in OC. Aberrant expression of the lncRNAs depicted on top (green circles) leads to taxane resistance through three main mechanisms: cell cycle regulation, upregulation of drug transporters leading to efflux of the drug, or epithelial-mesenchymal transition. The molecular mechanisms linking the lncRNAs to these resistance mechanisms involve interactions with miRNAs (light blue) and regulation of transcription factors or signaling pathways (black), as illustrated above.

A list of lncRNAs associated with taxane resistance in OC is detailed in Table 2. Few of these lncRNAs have well-described functions and will be presented below. The interplay between the described lncRNAs, their molecular pathways, and resistance mechanisms are illustrated in Figure 4.

**Table 2:** List of lncRNAs associated with taxane-resistance in ovarian cancer.

**lncRNA**	**Category**	Expression in OC tissue^*^	Expression in paclitaxel-resistant cell lines^**^	Mechanisms of resistance^***^	**Reference**
*UCA1*	Intergenic	N/A	↑	↓ miR-129 → ↑ abcb1	(155, 156)
*FER1L4*	Pseudogene	↓	↓	MAPK	(177)
*LINC01118*	Intergenic	↑	↑	↓ miR-134 → ↑ abcc1	(178)
*NEAT1*	Intergenic	↑ (paclitaxel resistance)	↑	↓ miR-194 → ↑ ZEB1	(179)
*Xist*	Intergenic	↓ (recurrent disease)	↓	N/A	(180)
*KB-1471A8.2*	Antisense	↓	↓	↓ CDK4	(181)
*OIP5-AS1*	Antisense	N/A	↓	N/A	(182)

*The order of the lncRNAs corresponds to the appearance of the individual descriptions in the manuscript. Only the first 4 lncRNAs are described in detail in the manuscript and are selected based on substantiating evidence in the literature*.

* *The expression of the lncRNAs in OC tissue is indicated by arrows, ↑ for higher and ↓ for lower expression in resistant patients (patient characteristics are indicated in parenthesis), compared to expression in sensitive patients. If no patient characteristics are indicated, the expression was determined in ovarian cancer tissue from patients with unspecified sensitivity to platinum drugs and normalized to adjacent or normal ovarian tissue*.

** *The expression in paclitaxel-resistant OC cell lines is indicated by arrows; ↑ for higher and ↓ for lower expression and the drug they are resistant to is indicated in parenthesis*.

*** *The effect of lncRNAs on associated pathways, miRNAs, genes or transcription factors involved in resistance mechanisms are indicated by arrows: ↑ induction and ↓ repression*.

*N/A, information not available*.

The Fer-1-like family member 4 (FER1L4) pseudogene is a lncRNA associated with tumor-suppressive properties in cancer (183). *FER1L4* acts as a decoy for miR-106a-5p which also interacts with the tumor suppressor *PTEN* (184). In OC, *FER1L4* is expressed at low levels compared to normal ovarian epithelial cells and, at even lower levels in paclitaxel-resistant cell lines. Transfection with *FER1L4* led to MAPK pathway suppression and restored the sensitivity to paclitaxel, indicating an important role in the development of resistance (177). Overall, PTEN-AKT-mTOR and MAPK are major cancer driver pathways deeply involved in resistance to chemotherapy (including paclitaxel) in several cancers (185).

Long Intergenic Non-Coding RNA 1118 (LINC01118) was recently identified as overexpressed in paclitaxel and cisplatin-resistant cell lines and OC compared with normal and benign tissues (178). These findings were supported by *in vitro* studies showing that knockdown conferred increased sensitivity to paclitaxel, whereas overexpression led to resistance. MiR-134 was predicted as a direct target, and functional assays showed that *LINC01118* was able to regulate the *ABCC1* gene through miR-134 repression (178). As previously described, *ABCC1* upregulation is associated with multidrug resistance in cancers (132). No additional studies have been performed correlating *LINC01118* with drug resistance or even cancer, warranting further investigations of this lncRNA.

Nuclear paraspeckle assembly transcript 1 (NEAT1) is transcribed from the *MEN1* (familial tumor syndrome multiple endocrine neoplasia type 1) locus on chromosome 11 and is a well-described oncogene (186). *NEAT1* is overexpressed in OC tissue and cell lines accordingly and is correlated with metastatic potential and poor prognosis (79, 187). In paclitaxel-resistant OC cells, *NEAT1* acts as a decoy for miR-194, promoting upregulation of *ZEB1* (zinc finger E-box-binding homeobox 1) (179). *ZEB1* is an important transcription factor and mediator of EMT and was previously associated with drug resistance (188). Besides, paclitaxel resistance was attenuated by *NEAT1* knockdown, which was associated with suppression of the efflux pump P-glycoprotein encoded by the *ABCB1* gene. This effect was also related to the interaction between *NEAT1* and miR-194, since the suppression of the efflux pump was rescued by miR-194 knockdown. The involvement of *NEAT1* in resistance to paclitaxel was validated in OC xenografts in mice, where knockdown restored paclitaxel sensitivity (179). These results substantiate the role of *NEAT1* in paclitaxel resistance and indicate the potential for therapeutic targeting.

## Perspectives and Future Directions

Collectively, the present review provides compelling evidence of the association between lncRNA expression pattern and therapeutic response in OC, indicating that the etiology of acquired resistance is more complex than originally described. We are just beginning to understand the biological role and function of some of these lncRNAs, and how they can be exploited for clinical purposes.

Two of the most obvious applications for lncRNAs in OC is the establishment of biomarker panels with predictive value for prognosis and/or drug response, or for therapeutic targeting to prevent or reverse resistance to chemotherapy. The presence of circulating lncRNAs in body fluids such as blood and urine at detectable levels, suggests that they could represent excellent biomarkers (189, 190). Several lncRNA signatures with predictive value for platinum-sensitivity in OC have recently been identified (49, 114, 120). However, further studies are needed to determine their clinical applicability.

The oncogenic behavior of some lncRNAs, combined with their tissue specificity and content of targetable residues, emphasize their potential as targets for therapeutic intervention. Furthermore, lncRNA targeting is one of the only therapeutic approaches to upregulate tumor suppressors in a locus-specific manner (191). Artificially synthesized polymers of nucleic acids, known as peptide nucleic acids (PNAs) are thermally stable, not affected by nucleases and can be modified for *in vivo* administration. Also, PNAs specific for RNA targets is much easier to design and synthesize than small-molecule oncogene inhibitors (192). For example, HOTAIR was targeted with a PNA, designed to prevent the interaction between *HOTAIR* and the EZH2 subunit of PRC2, in mice with platinum-resistant ovarian tumor xenografts. The treatment reduced *HOTAIR* expression and re-sensitized the tumors to treatment with cisplatin, which resulted in prolonged survival. The study provided proof of concept for targeting oncogenic lncRNAs as a strategy for precision medicine (193). However, more conclusive evidence of the complex molecular interactions of individual lncRNAs is paramount to determine the physiological impact of targeted treatments before clinical testing.

So far, the molecular profiling of lncRNAs and the identification of functional interactions have proved to be difficult to replicate in different studies. The main platforms for high throughput analysis of lncRNAs, such as RNA sequencing and expression arrays, offer different advantages and drawbacks, and the downstream bioinformatics is not yet standardized. Although RNA sequencing offers the advantage of including all potential lncRNA transcripts, the complexity of the following sequence assembly often hampers the correct annotation (194). In contrast, array-based methods provide a more standardized workflow and a much simpler downstream analysis but are limited to a selection of annotated transcripts. Several studies revise old data sets from publically available sources to perform *in silico* investigations. However, the experimental setup behind these data sets was rarely designed for the identification of lncRNAs. The low expression of lncRNA transcripts requires specific methodological considerations for optimal results. Furthermore, computational prediction of functional interactions should always be validated experimentally in the specific tissue of interest.

The studies investigating lncRNAs are increasing exponentially and both, experimental and bioinformatic methods are constantly improving. Several lncRNA-targeting therapeutics are already in the clinical pipeline (191), and some have reached clinical trials (195, 196). GENCODE (23), a spin-off from ENCODE is currently attempting to annotate all non-coding transcripts of the entire human genome. Complete annotation of human lncRNAs, standardization of experimental procedures and bioinformatic analysis combined with improved insights into the functional roles of lncRNAs in the development of resistance, will provide a novel paradigm for biomarker discovery and precision medicine in OC.

## Author Contributions

CA and SR conceived and designed the study. CA drafted the manuscript and designed the figures. LD and KS participated in drafting the molecular and clinical information, respectively. SR revised and edited the full content of the manuscript. All authors have read, revised critically and approved the final version of the manuscript.

### Conflict of Interest

The authors declare that the research was conducted in the absence of any commercial or financial relationships that could be construed as a potential conflict of interest.
